# Social determinants of psychological distress in Sierra Leone

**DOI:** 10.1007/s00127-022-02278-y

**Published:** 2022-04-19

**Authors:** Kanykey Jailobaeva, Rebecca Horn, Stella Arakelyan, Karin Diaconu, Ajaratu Kamara, Alastair Ager

**Affiliations:** 1 NIHR Global Health Research Unit on Health in Situations of Fragility, Institute for Global Health and Development, Queen Margaret University, Edinburgh, EH21 6UU UK; 2 College of Medicine and Allied Health Sciences, University of Sierra Leone, Freetown, Sierra Leone

**Keywords:** Social determinants, Psychological distress, Mental health, Sierra Leone, Fragile setting

## Abstract

**Purpose:**

Growing evidence demonstrates that daily stressors such as family violence, unemployment, and living conditions play an important part in causing psychological distress. This paper investigates the impact of distressing events and day-to-day living conditions on psychological distress in the fragile context of Sierra Leone.

**Methods:**

A cross-sectional survey was conducted with 904 adults (454 men, 450 women) in 5 districts of Sierra Leone. The survey questionnaire comprised the Sierra Leone Psychological Distress scale and measures of demographic variables and personal characteristics, current life circumstances and potentially distressing events.

**Results:**

Multiple regression results identified three factors to be the greatest contributors to psychological distress: family conflict (*β* = 0.185, *p* < 0.001) and inability to afford basic needs (*β* = 0.175, *p* < 0.001). Gender differences were evident: factors predicting men’s psychological distress included severe sickness or injury (*β* = 0.203, *p* < 0.001) and being unable to afford basic needs (*β* = 0.190, *p* < 0.001); for women, predicting factors were family conflict (*β* = 0.212, *p* < 0.001), perceived poor health (*β* = 0.192, *p* < 0.001) and inability to afford basic needs (*β* = 0.190, *p* < 0.001).

**Conclusion:**

Initiatives to promote good mental health and psychosocial wellbeing in Sierra Leone should focus on enhancing income-generating and employment opportunities, promoting access to education, and strengthening family relationships.

**Supplementary Information:**

The online version contains supplementary material available at 10.1007/s00127-022-02278-y.

## Introduction

There is now a solid body of evidence which demonstrates that daily stressors and living conditions have as much, if not more, impact on psychological distress than experiences of potentially traumatic events [1–6]. Daily stressors commonly identified as predicting poor mental health outcomes include family violence, unemployment, perceived discrimination, food insecurity and poverty, together with broader factors such as unequal access to basic resources and opportunities to partake in occupational and recreational activities [7–9].

Attempts to address mental health concerns must, therefore, attend to the factors of day-to-day life that contribute to distress to prevent mental health problems and promote mental health. This is an important corrective to the tendency to focus mental health resources on in-patient psychiatric provision and other forms of specialist mental health care. More than 80% of public expenditure on mental health in low-income countries has been found to be allocated to psychiatric hospitals [10]. The recent Lancet Commission on Global Mental Health and Sustainable Development [11] advocates for an expanded agenda for mental health that addresses promotion and prevention as well as treatment and rehabilitation, noting that the greatest population benefit is gained from promoting factors that facilitate good mental health and avoiding causes of ill-health.

In contexts where resources (both financial and human) for mental health provision are limited, it is especially important to focus on prevention and promotion to reduce the numbers requiring specialist mental health care and ensure that those who need such services are able to access them [12].

### Sierra Leone

Sierra Leone is a low-income country in Western Africa with a population of over seven million people [1]. Development challenges, including poor governance, unemployment, limited resources [2], a history of civil war between 1991 and 2002 [3], an Ebola outbreak in 2014 [4], and a natural disaster resulting in mudslide and flooding in 2017 [5] contribute to the fragility of the country.

Studies on mental health in Sierra Leone have largely focused on the impact of extreme events, notably the civil war and the Ebola outbreak, on the psychological wellbeing of various groups in the population, such as children involved with armed groups [6–8] and Ebola survivors [9, 10]. There is little research on psychological distress and its determinants in the general adult population outside of an emergency context.

Formal mental health service provision in Sierra Leone is limited to one psychiatric hospital in the capital city, Freetown, which receives referrals from provincial and district hospitals, NGO services, and recent attempts to strengthen capacity at primary care level through the training of mental health nurses [13].

### Aims of this study

In an earlier stage of this project, we investigated the problems that adults in Sierra Leone said affected the lives of women and men in their community, using a socio-ecological framework to structure their responses [14]. Overall, respondents located problems predominantly at community and societal levels. Women identified significantly more problems at the family level than men, particularly related to relationships with an intimate partner, and men identified significantly more problems at the societal level than women, primarily related to lack of infrastructure. Men and women were equally focused on problems related to poverty and lack of income-generating opportunities. Poverty and inability to earn an income underpinned many of the problems described at individual, family, and community levels.

Subsequently, we developed a measure of psychological distress specifically for the Sierra Leone context [15] (Psychology paper). The study reported here brought these two elements of our work together to investigate the impact of distressing events and day-to-day living conditions on psychological distress in Sierra Leone.

## Methods

### Study design and sample

A cross-sectional survey of adults (> 18) was conducted in five districts of Sierra Leone (Kailahun, Bo, Kono, Kambia, and Western Area), which were purposefully selected to represent the geographical, economic, and cultural diversity of the country. Within each district, five chiefdoms were selected using a Probability Proportional to Size (PPS) strategy [16]. In each chiefdom, six villages were randomly selected. In each village, six households were surveyed using a "random walk" strategy [17]. We used a grid by de Vaus to select individuals within the households [18]. First, individuals aged over 18 years old in the selected household were listed from eldest to youngest and assigned a number from 1 to *N*. Referring to the grid, enumerators then selected a person based on the order number of the household that the enumerator was surveying for that data and the number of eligible people in the household.

Since the survey was conducted to validate a new measure of psychological distress for Sierra Leone [15], our sample size estimations were guided by the literature on determining the sample size for factor analyses [19–21]. With reference to Mundfrom and colleagues [21], we envisaged our sample to have a variables-to-factors ratio of four, wide communality (between 0.2 and 0.8), and excellent coefficient congruence (*K* value 0.98). Based on these criteria, our target sample size was 900 [21]**.** With an anticipated non-response rate of 20%, we targeted 1100 households. In practice, enumerators approached 1344 households, 904 of which participated in the survey. The survey was conducted electronically using Open Data Kit on tablets.

### Survey questions

The survey questionnaire contained the Sierra Leone Psychological Distress (SLPD) scale to measure psychological distress. This scale was developed and validated in a three-phase mixed-method exploratory sequential study to produce a locally appropriate measure of psychological distress for Sierra Leone [15]. The final SLPD scale consisted of 18 items and showed good internal consistency (Cronbach's alpha = 0.89). The scale has three subscales with good internal consistency (Cronbach's alpha greater than 0.7). The first subscale consists of eight items around high emotional arousal and labile mood. Five items in the second subscale focus on hopelessness, withdrawal, and feelings of worthlessness. The final third subscale contains five items on negative feelings and changes in behaviours indicating wellbeing (sleep, appetite, social behaviours). Survey respondents were asked to indicate how much they had had experiences corresponding to the items in the SLPD scale over the last 1 week. Response choices included "Not at all" (0), "A little" (1), "Quite a lot" (2), and "Very much" (3).

The survey tool also included questions on factors which had been identified in previous stages of the project as potentially contributing to distress [14]. These included (a) standard background variables (gender, age, ethnicity, education, religion, household size), (b) individual characteristics (marital status, responsibilities, financial situation, health status, religious practice, and literacy), (c) life circumstances, such as a lack of support, safety, and access to facilities and services, and (d) potentially distressing events such as the end of a marriage or relationship and severe sickness.

For marital status, respondents were asked to indicate whether they were: never married, separated/divorced/widowed, married/living with a partner where both partners did not have any other relationships (single-partner relationship), and married/living with a partner where at least one of the partners had another relationship (multiple-partner relationship). To measure responsibilities for dependants under 18 years old, respondents were asked how many biological and non-biological children they were responsible for.

Perception of financial situation was measured by asking respondents to rate on a five-point Likert scale how well they were managing financially right now. Respondents were also asked if they had any job/work paid in cash or kind either as an employee or self-employed. Literacy was measured by asking whether they could read well enough to read a book or a newspaper. Regarding health, respondents were asked to rate on a four-point Likert scale their overall health and how much physical health problems had limited their daily work over the last 1 month. Respondents also indicated regularity of their attendance at religious services/prayers based on these options: at least once most weeks, once or twice a month, less than once a month, not at all/only for burials or weddings.

To measure support available in challenging situations, respondents were asked how confident they were on a four-point Likert scale that they could (i) talk to someone they trust in their community about their concerns and (ii) borrow money from a friend or family member so they could help their child or family member. To assess safety, respondents were asked on a three-point Likert scale if their community was safe to walk around (i) during the daytime and (ii) during the night. Respondents were also asked to rate on a three-point Likert scale their access to community infrastructure comprising: an adequate water supply, an adequate rubbish disposal system, an adequate electricity supply, adequate toilets, adequate road network and transportation, adequate community centres and halls, adequate recreational facilities, places of worship, adequate schools, adequate vocational training opportunities, and adequate health care centres. Finally, respondents were asked to rate on a four-point Likert scale how much they had been disturbed in the last month by loss/death of a loved one, loss of property or money, severe sickness or injury, family conflict, disappointment in job/college/business, someone using physical violence towards you, being unable to afford basic needs, and end of a marriage or relationship.

### Procedure

The data collection team consisted of ten enumerators (five males and five females). All enumerators were Sierra Leoneans, aged between 20 and 30 years old, represented a range of ethnic groups, and spoke local languages, including Mende, Temne, Fullah, Limba, Krio, and English. Researchers from [institution blinded for peer review] provided enumerators with 5-day training before fieldwork that included intensive practical training in the measures and methods. Staff members from the College of Medicine and Allied Health Sciences (COMAHS), University of Sierra Leone, coordinated logistical issues. Researchers from [institution blinded for peer review] and staff from COMAHS provided continuous supervision and support to enumerators during the fieldwork.

### Ethical consideration

This study was approved by [institution blinded for peer review] Research Ethics Committee and by the Office of the Sierra Leone Ethics and Scientific Review Committee, Ministry of Health and Sanitation. A standard information sheet about the survey was read to respondents who gave verbal informed consent to take part in the survey. The enumerators documented the verbal consent of respondents. Names of survey participants were not recorded.

### Data analysis

Data were analysed using IBM SPSS version 23. Descriptive analysis was run for demographic variables. Independent t tests for continuous variables and chi-square tests for categorical variables were performed to detect statistically significant differences in the responses of male and female respondents. Composite scores for the SLPD scale and its subscales were estimated, with higher scores suggesting higher distress. Composite scores for social connectedness, perceived safety, and community infrastructure were derived by summing responses, but with reverse coding such that high scores indicated negative values, i.e., lacking the confidence to ask help from family and friends, lacking a sense of safety, and lacking access to adequate infrastructure. We used a Kruskal–Wallis test to identify statistically significant gender differences in the composite scores for the SLPD scale, its subscales, social connectedness, perceived safety, access to community infrastructure, and distress events. Univariate linear regressions were performed for each independent variable to exclude those with a *p* value less than 0.1 from further analysis. A multicollinearity test identified no collinearity in data as all VIF values were below ten, and all tolerance statistics were above 0.2 [22] (Annex 1 in Supplementary Information). A stepwise approach was used for developing final predictive models. Multiple linear regressions were run for the SLPD scale and its subscale for the entire sample and disaggregated by gender.

## Results

### Sample characteristics and perceptions on health status

Table 1 presents the sample characteristics. The sample was gender-balanced (*n* = 450, 49.8% men and *n* = 454, 50.2% women). The median age of respondents was 37 years: 39 years for men (SD = 17.33) and 35 years for women (SD = 15.58). Mende (*n* = 327, 36.2%), Temne (*n* = 154, 16%), and Kono (*n* = 145, 17%) were the largest ethnic groups in the sample. There were more Muslims (*n* = 615, 68%) than Christians (*n* = 286, 31.6%). A majority of participants (*n* = 729, 80.6%) attended religious services/prayers frequently.

**Table 1:** Sample characteristics

Variables	Total sample (*n* = 904)		Males (*n* = 450)		Females (*n* = 454)	
Age (years)						
18–29	291	32.2%	135	30.0%	156	34.4%
30–39	204	22.6%	101	22.4%	103	22.7%
40–49	158	17.5%	85	18.9%	73	16.1%
50–59	118	13.1%	58	12.9%	60	13.2%
60–69	77	8.5%	32	7.1%	45	9.9%
70<	56	6.2%	39	8.7%	17	3.7%
Ethnic groups						
Mende	327	36.2%	165	36.7%	162	35.7%
Temne	154	17.0%	72	16.0%	82	18.1%
Kono	145	16.0%	72	16.0%	73	16.1%
Other	278	30.8%	141	31.3%	137	30.1%
Religion						
Christian	286	31.6%	139	30.9%	147	32.4%
Muslim	615	68.0%	311	69.1%	304	67.0%
Other	3	0.3%	-	-	3	0.7%
Religious practice						
Less frequent (between once or twice a month to not at all/only for burials and weddings)	175	19.4%	80	17.7%	95	20.9%
Frequent (at least once most weeks)	729	80.6%	370	82.2%	359	79.1%
Marital status						
Never married	138	15.2%	79	17.6%	59	13.0%
Separated/divorced/widowed	156	17.3%	45	10.0%	111	24.4%
Married/living with partner—(single-partner relationship)	454	50.2%	291	64.7%	163	35.9%
Married/living with partner—(multiple-partner relationship)	146	16.2%	30	6.6%	116	25.6%
Household size (individuals)						
1–5	314	34.7%	165	36.7%	149	32.8%
6–10	451	49.9%	217	48.2%	234	51.5%
11<	139	15.4%	68	15.1%	71	15.6%
Responsible for dependants aged > 18						
No dependents	98	10.9%	69	15.3%	29	6.4%
1–5	265	29.3%	115	25.6%	150	33.0%
6–10	313	34.6%	152	33.8%	161	35.5%
11<	228	25.2%	114	25.3%	114	25.1%
Education						
No education	392	43.4%	149	33.1%	243	53.5%
Primary only	140	15.5%	76	16.9%	64	14.1%
Above primary	372	41.1%	225	50.0%	147	32.4%
Literacy level						
Can read	337	37.3%	214	47.6%	123	27.1%
Cannot read	567	62.7%	236	52.7%	331	72.9%
Employment status						
Employed	534	59.1%	307	68.2%	227	50%
Unemployed	370	40.9%	143	31.8%	227	50%
Managing financially						
No difficulty at all	175	19.4%	119	26.4%	56	12.3%
A little bit of difficulty	230	25.5%	134	29.8%	96	21.1%
A moderate amount of difficulty	162	17.9%	84	18.7%	78	17.2%
A lot of difficulty	236	26.1%	88	19.6%	148	32.6%
Not managing at all	101	11.1%	25	5.5%	76	16.8%
General health						
Very bad	46	5.1%	9	2.0%	37	8.1%
Bad	205	22.7%	100	22.2%	105	23.1%
Good	508	56.2%	249	55.3%	259	57.1%
Excellent	145	16.0%	92	20.5%	53	11.7%
Physical health problems limiting daily work						
Not at all	493	54.5%	262	58.2%	231	50.9%
A little	223	24.7%	112	24.9%	111	24.4%
Quite a bit	121	13.4%	54	12.0%	67	14.8%
Extremely	67	7.4%	22	4.9%	45	9.9%

Two-thirds of respondents (*n* = 600, 66.4%) reported being in a relationship. A significantly higher proportion of men (*n* = 321, 72%) reported being in a relationship than women (*n* = 279, 62%), *χ*^2^ (4) = 117.55, *p* < 0.001. The average household size was 7 (SD = 4.02). Half of the respondents (*n* = 451, 50%) indicated having between 6 and 10 people in the household. On average, respondents reported being responsible for eight dependants under 18 years (SD = 6.00).

Forty-three percent of the sample reported no formal education, with women being significantly less educated than men, *χ*^2^ (2) = 39.91, *p* < 0.001. In terms of literacy, 63% of respondents (*n* = 567) reported not being able to read a book or newspaper, with women again reporting significantly lower rates, *χ*^2^ (1) = 40.47, *p* < 0.001. Only 19% of the sample (*n* = 175) said they did not have any difficulty managing financially. The remaining 81% (*n* = 729) had some level of difficulty. Women reported significantly more difficulty managing financially than men (*t* (902) = − 8.63, *p* < 0.001).

Nearly, one-third (*n* = 251, 28%) of the sample reported having either very poor or poor health, with women having significantly worse health than men (*t* (902) = − 4.45, *p* < 0.001). Slightly more than half (*n* = 493, 55%) of respondents reported having no physical health problems limiting their daily work. The daily work of the remaining 45% was limited by their physical health problems to various extents, with women being more significantly limited than men (*t* (902) = − 3.20, *p* < 0.001).

### Factors potentially contributing to distress

Respondents reported being moderately confident to talk to a trusted person about their concerns (mean 1.46, SD = 1.12) and borrow money from a friend/family for family needs (mean 1.68 of 3, SD = 1.12). They also reported having moderate access to community infrastructure (13.26 of 22, SD = 4.60) and felt safe in their communities (0.63 of 4, SD = 0.93). Unable to afford basic needs had the highest mean (mean 1.62, SD = 1.05) among events contributing to distress (Table 2).

**Table 2:** Distress factors disaggregated by gender

Factor	Total sample		Males		Females	
	Mean	SD	Mean	SD	Mean	SD
Lack of confidence to talk to a trusted person about concerns (max score—3)	1.46	1.12	1.38	1.10	1.54*	1.13
Lack of confidence to borrow money from a friend/family for family needs (max score—3)	1.68	1.12	1.54	1.15	1.83***	1.07
Lack of community safety (max score–4)	0.63	0.93	0.44	0.84	0.82***	0.98
Lack of community infrastructure (max score–22)	13.26	4.60	13.09	4.67	13.43	4.53
Loss (death) of a loved one (max score—3)	0.89	1.21	0.83	1.14	0.95	1.28
Loss of property or money (max score—3)	0.61	1.02	0.48	0.92	0.74***	1.10
End of a marriage or relationship (max score—6)	0.79	1.42	0.59	1.22	0.98***	1.56
Severe sickness or injury (max score—3)	0.56	0.94	0.52	0.91	0.60	0.97
Family conflict (max score—3)	0.47	0.91	0.33	0.74	0.60***	1.05
Disappointment in job/ college/ business (max score—3)	0.86	1.03	1.04	1.08	0.68	0.94
Someone using physical violence towards you (max score—3)	0.13	0.51	0.06	0.34	0.20***	0.63
Being unable to afford basic needs (max score—3)	1.62	1.05	1.62***	1.02	1.62	1.08

*p < 0.05, **p < 0.01, ***p < 0.001

Some factors affected women significantly more than men. Women reported feeling significantly less confident to talk to a trusted person about concerns (H(1) = 4.621, *p* < 0.05) and borrow money from a friend/family for family needs (H(1) = 14.92, *p* < 0.001). Women also felt less safe in the community (H(1) = 39.25, *p* < 0.001). Women had been significantly more disturbed than men over the previous 1 month by loss of property or money **(**H (1) = 14.43, *p* < 0.001), end of a marriage or relationship (H(1) = 14.27, *p* < 0.001), family conflict (H(1) = 12.80, *p* < 0.001), and someone using physical violence towards them (H(1) = 14.58, *p* < 0.001). Men had been more significantly disturbed than women over the previous month by disappointment related to a job/college/business (H (1) = 26.76, *p* < 0.001) (Table 2).

### Psychological distress

Table 3 shows the scores for the full SLPD scale and its subscales disaggregated by gender. A mean SLPD score for all respondents was 13.16 of 54 (SD 9.44). The subscale scores were 7.48 of 24 (SD 5.00) for high emotional arousal and labile mood, 1.86 of 15 (SD 2.79) for hopelessness, withdrawal, and worthlessness, and 3.82 of 15 (SD 3.22) for negative feelings and changes in behaviour. Women had significantly higher scores for the overall psychological distress and its subscales (H(1) = 57.28, *p* < 0.001 for the SLPD scale, H(1) = 23.67, *p* < 0.001 for Subscale 1, H(1) = 40.11, *p* < 0.001 for Subscale 2, H(1) = 83.53, *p* < 0.001 for Subscale 3).

**Table 3:** Multiple regression outcomes

Variables	Overall psychological distress (Model 1)	High emotional arousal and labile mood (Model 2)	Hopelessness, withdrawal, and worthlessness (Model 3)	Negative feelings and changes in behaviour (Model 4)
	*β*	*β*	*β*	*β*
Family conflict	0.185***	0.162***	0.108***	0.200***
Being unable to afford basic needs	0.175***	0.169***	0.166***	0.135***
Perceiving health as poor	0.169***	0.105**	0.139***	0.143***
End of a marriage or relationship	0.162***	0.168***	0.163***	0.066*
Severe sickness or injury	0.149***	0.099**	0.095**	0.168***
Gender (ref male)	0.134***	0.068*	0.064*	0.208***
Loss/death of a loved one	0.109***	0.121***	0.085**	0.067*
Disappointment in job/college/business	0.113***	0.136***	0.103***	
Lack safety in the community	0.060*	0.062*	0.106***	
Perceived difficulty to manage financially	0.088**		0.143***	0.087**
Loss of property or money	0.063*	0.075**		
Physical health problems limiting daily work		0.101**		
Lack of confidence to talk to someone trustful about concerns			0.082**	
Cannot read (ref can read)			0.115***	
Marital status (ref married/living with partner—(multiple-partner relationship))				
Never married			0.061*	0.86**
Separated			0.058	0.072*
Married/living with partner—(single-partner relationship)			0.028	0.008
*R*^2^	0.433	0.353	0.342	0.337
*F* (df 1;2)	61.869*** (11; 892)	44.201*** (11; 982)	35.150*** (13; 880)	44.885*** (10; 883)

*p < 0.05, **p < 0.01, ***p < 0.001

### Predictors of distress

Table 3 shows the results of the multiple regression models. Family conflict (*β* = 0.185, *p* < 0.001) and being unable to afford basic needs (*β* = 0.175, *p* < 0.001) were the strongest predictors of distress (Model 1). This model explained 43% (*R*^2^ = 0.433) of the variance in the distress score, *F* (11;892) = 61.87, *p* < 0.001. High emotional arousal and labile mood (Model 2) were predicted most by being unable to afford basic needs (*β* = 0.169, *p* < 0.001) and end of a marriage or relationship (*β* = 0.168, *p* < 0.001). The model accounted for about a quarter (35%, *R*^2^ = 0.353) of the variance in the score on high emotional arousal and labile mood, *F* (11; 982) = 44.20, *p* < 0.001. Being unable to afford basic needs (*β* = 0.166, *p* < 0.001) and end of a marriage or relationship (*β* = 0.163, *p* < 0.001) were also strongly predicted with hopelessness, withdrawal, and worthlessness (Model 3). Around a quarter (34%, *R*^2^ = 0.342) of the variance in the score on hopelessness, withdrawal, and worthlessness was explained by this model, *F* (13; 880) = 35.15, *p* < 0.001. Finally, negative feelings and changes in behaviour (Model 4) were strongly predicted by gender (*β* = 0.208, *p* < 0.001) and family conflict (*β* = 0.200, *p* < 0.001). This model explained 34% (*R*^2^ = 0.337) of the variance in the score on negative feelings and changes in behaviour, *F* (10; 883) = 44.89, *p* < 0.001.

### Predictors of distress by gender

**Fig. 1:**
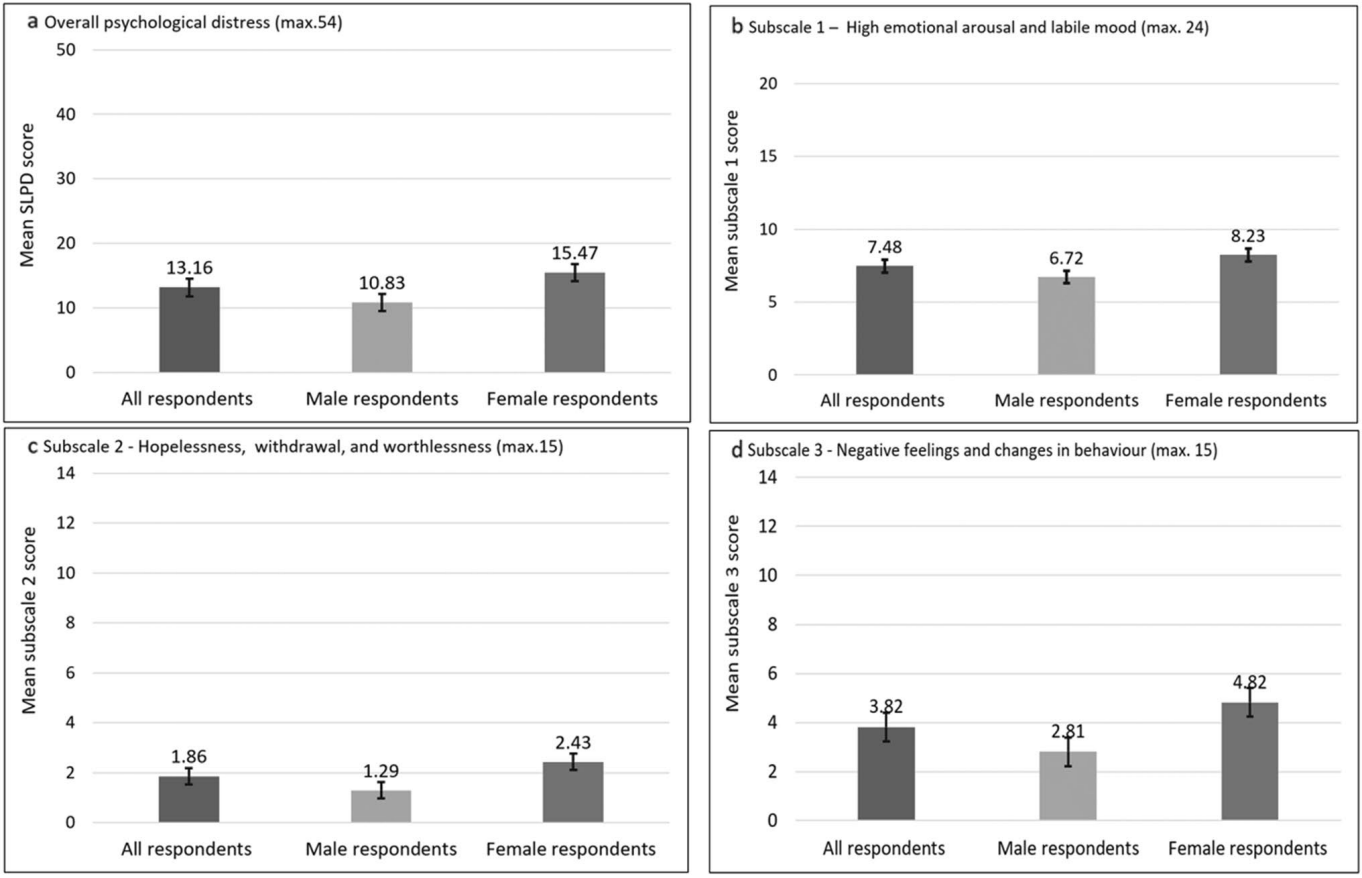
Scores of the SLPD scale and its subscales for the whole sample and broken down by gender

Considering significant differences in the distress scores of male and female participants (Fig. 1), separate multiple regressions were conducted for these two groups. Table 4 presents the results of the multiple regression for male participants. The overall psychological distress of male respondents (Model 1) was strongly predicted by severe sickness or injury (*β* = 0.203, *p* < 0.001) and being unable to afford basic needs (*β* = 0.190, *p* < 0.001). The model explained 36% (*R*^2^ = 0.364) of the variance in the psychological distress of men, *F* (9; 435) = 27.70, *p* < 0.001. Men’s score on high emotional arousal and labile mood (Model 2) was also strongly predicted with being unable to afford basic needs (*β* = 0.203, *p* < 0.001) and severe sickness or injury (*β* = 0.172, *p* < 0.001). This model accounted for around about a quarter (34%, *R*^2^ = 0.336) of the variance in the male’s score on high emotional arousal and labile mood, *F* (9; 440) = 27.77, *p* < 0.001. Being unable to read (*β* = 0.204, *p* < 0.001) and having physical health problems which limit daily work (*β* = 0.184, *p* < 0.001) were the leading predictors of men’s score on hopelessness, withdrawal, and worthlessness (Model 3). Twenty-six percent (*R*^2^ = 0.255) of the variance in this score of male participants was explained by this model, *F* (9; 435) = 16.50, *p* < 0.001. The score of male respondents on negative feelings and changes in behaviour (Model 4) were strongly predicted by perceiving health as poor (*β* = 0.219, *p* < 0.001) and severe sickness or injury (*β* = 0.173, *p* < 0.001). The model explained 28% (*R*^2^ = 0.281) of the variance in this score of male participants, *F* (7; 442) = 24.69, *p* < 0.001.

**Table 4:** Multiple regression outcomes based on male participants

Variables	Overall psychological distress (Model 1)	High emotional arousal and labile mood (Model 2)	Hopelessness, withdrawal, and worthlessness (Model 3)	Negative feelings and changes in behaviour (Model 4)
	*β*	*β*	*β*	*β*
Family conflict	0.157***	0.131***	0.096*	0.160***
Disappointment in job/college/business	0.140***	0.138***	0.105**	0.163***
Severe sickness or injury	0.203***	0.172***		0.173***
Perceiving health as poor	0.160***	0.121**		0.219***
Loss/death of a loved one	0.106**	0.102**		0.131**
Being unable to afford basic needs	0.190***	0.203***	0.165***	
End of a marriage or relationship	0.135***	0.138***	0.145***	
Perceived difficulty to manage financially	0.086*		0.155***	0.129**
Marital status (ref married/living with partner—(multiple-partner relationship))				
Never married	0.019		0.019	
Separated	0.016		0.023	
Married/living with partner—(single-partner relationship)	− 0.101**		− 0.105*	
Religious practice (ref less frequent)		− 0.097**		
Loss of property or money		0.008*		
Cannot read (ref can read)			0.204***	
Physical health problems limiting daily work			0.184***	
Lack of confidence to talk to someone trustful about concerns			0.133**	
Lack of community infrastructure				− 0.135***
*R*^2^	0.364	0.336	0.255	0.281
*F* (df 1;2)	27.704*** (9; 435)	24.772*** (9; 440)	16.502*** (9; 435)	24.692*** (7; 442)

*p < 0.05, **p < 0.01, ***p < 0.001

Table 5 shows outcomes of the multiple regression for female participants. Family conflict (*β* = 0.212, *p* < 0.001), perceived poor health (*β* = 0.192, *p* < 0.001) and inability to afford basic needs (*β* = 0.190, *p* < 0.001) strongly predicted women’s overall psychological distress (Model 1). Forty-three percent (*R*^2^ = 0.432) of the variance in the SLPD scores of women were explained by this model, *F* (10; 443) = 33.66, *p* < 0.001. High emotional arousal and labile mood of women (Model 2) were strongly predicted with family conflict (*β* = 0.202, *p* < 0.001) and end of a marriage or relationship (*β* = 0.194, *p* < 0.001). This model accounted for 35% (R^2^ = 348) of variance in the score of female participants on high emotional arousal and labile mood, *F* (8; 445) = 29.64, *p* < 0.001. Perceiving health as poor (*β* = 0.213, *p* < 0.001) and being unable to afford basic needs (*β* = 0.198, *p* < 0.001) had the largest effect size on women’s score on hopelessness, withdrawal, and worthlessness (Model 3). This model explained 39% (*R*^2^ = 0.389) of the variance in this score of female participants, *F* (11; 442) = 25.62, *p* < 0.001. Finally, women’s scores on negative feelings and changes in behaviour (Model 4) were predicted most by family conflict (*β* = 0.227, *p* < 0.001) and being unable to afford basic needs (*β* = 0.172, *p* < 0.001). The model accounted for 28% (*R*^2^ = 0.282) of the variance in the women’s score on negative feelings and changes in behaviour *F* (8; 440) = 21.63, *p* < 0.001.

**Table 5:** Multiple regression outcomes based on female participants in the sample

Variables	Overall psychological distress (Model 1)	High emotional arousal and labile mood (Model 2)	Hopelessness, withdrawal, and worthlessness (Model 3)	Negative feelings and changes in behaviour (Model 4)
	*β*	*β*	*β*	*β*
Family conflict	0.212***	0.202***	0.108**	0.227***
Perceiving health as poor	0.192***	0.129*	0.213***	0.096*
Being unable to afford basic needs	0.190***	0.158***	0.198***	0.172***
End of a marriage or relationship	0.175***	0.194***	0.151***	0.092*
Loss/death of a loved one	0.113**	0.147***	0.081*	
Disappointment in job/college/business	0.096**	0.125**	0.133***	
Lack of safety in the community	0.089*	0.089*	0.146***	
Perceived difficulty to manage financially	0.089*		0.119**	0.099*
Severe sickness or injury	0.117**			0.169***
Loss of property or money	00.085*		0.081*	
Physical health problems limiting daily work		0.155**		
Employment (ref employed)			0.081*	
Lack of community infrastructure			0.087*	
Cannot read (ref can read)				− 0.113**
Marital status (ref married/living with partner—(multiple-partner relationship))				
Never married				0.031
Separated				0.099*
Married/living with partner–(single-partner relationship)				0.020
*R*^2^	0.432	0.348	0.389	0.282
*F* (df 1;2)	33.656*** (10; 443)	29.646*** (8; 445)	25.623*** (11; 442)	21.633*** (8; 440)

*p < 0.05, **p < 0.01, ***p < 0.001

## Discussion

The findings of this study highlight the differences in experiences of men and women in Sierra Leone. Female respondents reported being less educated and less literate than the male respondents and having poorer health. This is in line with other research which indicates lower levels of literacy for women than men in Sierra Leone and fewer girls than boys attending school [23]. Women also reported being less socially connected and feeling less safe in their communities. They were more likely than men to have been disturbed over the previous 1 month by the loss of property or money, by the end of a marriage or relationship, by family conflict or family violence. Men were more likely than women to have been disturbed by some kind of disappointment in their work or education.

The factors which were found to make the greatest contribution to psychological distress within the adult population of Sierra Leone were family conflict and inability to afford basic needs. High emotional arousal and labile mood (Subscale 1) were also predicted strongly by inability to afford basic needs and, additionally, by end of a marriage or relationship. These two variables were also the strongest predictors of hopelessness, withdrawal, and worthlessness (Subscale 2). Negative feelings and changes in behaviour (Subscale 3) were strongly predicted by gender and family conflict.

The factors which most strongly predicted psychological distress for men, across the SLPD scale as a whole and the subscales, were physical health issues (sickness and injury), financial issues and illiteracy. For women, the factors which had the greatest impact were inability to afford basic needs, poor health, the end of a marriage or relationship, and family conflict. This clearly demonstrates the gendered nature of life in Sierra Leone. Gender Concerns International report [24] that while many women (66%) in Sierra Leone are economically active, they do not record substantial growth in their economic activities compared to men, due to inadequate skills development, low educational status, low economic power, and restricted access to credit facilities. This often results in women’s economic dependency on men, which means that they are greatly affected in practical ways, as well as emotionally, when a relationship ends or when there is tension or conflict in their relationships. High rates of intimate partner violence have been reported in Sierra Leone, with 29% of ever-partnered women and girls aged 15–49 reporting having experienced physical and/or sexual violence by a current or former intimate partner in the previous 12 months [23]. Relationship problems, including domestic violence, family rejection, divorce, and poor parent–child interaction, have been shown in the literature to have a negative impact on mental health [25–29]. The findings of the current study also reinforce those reported from an earlier qualitative phase of this project [14], which found that women reported to a much greater extent than men that they were affected by problems at the family level. A greater proportion of men reported that they were affected by lack of income-generating opportunities. For men, the pressure to provide for their families in the absence of income-generating opportunities can contribute to tensions and conflict within the family, so affecting the wellbeing of all household members [30].

However, poverty was reported equally by men and women as a problem affecting their community in the earlier study, and these findings were supported by the current study, with ‘inability to afford basic needs’ as a strong predictor of psychological distress for both genders. There are high levels of poverty in Sierra Leone; UN Women report that 53% of the population live below the national poverty line, and 90% of the adult population experience severe food insecurity [23]. Studies in other low- and middle-income contexts have shown that individuals in socio-economically disadvantaged circumstances (e.g., low income, unemployment, food insecurity) are at increased risk of encountering mental health challenges [8, 31–34]. For example, food insecurity was identified as one of the key stressors in a study in Zambia [35]. Another example is the elderly in China whose depressive symptoms were connected to their limited social pension [36]. Poverty has consistently been found to be associated with increased sexual and gender-based violence in contexts including Sierra Leone [30, 37, 38]. Inequitable gender norms have been found to intersect with material resource scarcity to produce relational contexts in which women report limited agency over their relationships and financial independence [31]. This suggests a possible gendered interaction between the different factors identified in this study as contributing to distress.

There is likely to be a further interaction for men between physical health and inability to afford basic needs. Women reported poorer health than men, but physical health issues, including both injury and sickness, were only predictors of distress for male respondents. Whilst there is a growing literature indicating that problems with physical health can cause psychological distress [39], the fact that this was only the case for men in this study suggests that this may be linked to physical health problems constraining their ability to earn an income.

Given the circumstances of women in Sierra Leone, it is perhaps no surprise that they reported significantly higher levels of distress compared to men. Patel et al. refer to studies in various settings which have shown that ‘gender disempowerment interacts with other adversities such as poverty, gender-based violence, sexual harassment and food insecurity to increase the prevalence of common mental disorders in women’ [11].

### Implications of the findings

The findings of this study demonstrate that a strengthened economic situation, centred around enhanced income-generating opportunities, would make a particularly significant impact on mental health in Sierra Leone. Our findings reinforce those from other contexts in emphasising the importance of employment as a protective factor against mental disorders, especially for men [34, 40, 41]. Practical interventions on promoting access to education and employment opportunities have been shown to have positive effects on mental health [32, 34, 35]. Continued consultations with stakeholders in Sierra Leone at community level, leading to the development of locally relevant recommendations regarding economic strengthening, could build upon platforms already established in terms of the current development agenda in relation to, for example, the agriculture sector.

The majority of the rural population in Sierra Leone are engaged in agricultural activities, but the majority of these are small scale. Enhanced support for the agricultural sector in the country—though, for example, provision of seeds, improved technologies for planting and harvesting, and skills in improved farming techniques to enable farmers to move from subsistence farming to farming as a business—would be likely to have a positive impact on the wellbeing of many. This may apply particularly to those with lower levels of education.

Many of those with lower levels of education, including a large proportion of women, work in the informal economy, running small businesses. Access to loans with minimal interest rates would benefit these groups and enable people to engage in constructive activity that is likely to produce an income. The current interest rate is 20% at the government bank, and 22% at private banks, so small business owners risk accruing high levels of debt if their initiatives are unsuccessful. The strengthening of infrastructure, such as roads, bridges and ports, would support the movement of people and goods, so facilitating economic activity. A wide range of sectors has a role to play in promoting good mental health and psychosocial wellbeing [14].

Our findings also underline the role played by supportive family relationships in psychological wellbeing, especially for women. There are links between economic wellbeing and the quality of family relationships, and poverty has consistently been identified as a cause of family conflict [42, 43]. Practical interventions on family strengthening have been shown to have positive effects on mental health [44–46]. For example, Petersen and colleagues identified community-level parenting programmes during infancy as “best practice” to prevent mental health issues in low and middle-income countries [47].

Recent political initiatives to address the factors contributing to women’s distress in Sierra Leone include the Gender Equality and Women’s Empowerment policy, launched by the Minister of Gender and Children's Affairs in December 2020. This policy seeks to address gender inequalities, minimise poverty levels and incidences of social injustices, and enhance public and private investment to create a society in which all citizens have equal access to basic services and enjoy the same rights and opportunities in enabling environments. However, the embedded nature of harmful gendered norms creates challenges in achieving the aims of initiatives such as this.

### Limitations of the study

The subscales of the SLPD scale were developed based on the exploratory factor analysis. The confirmatory factor analysis is needed to confirm the proposed structure of subscales. The current study investigates the factors contributing to distress amongst the adult population of Sierra Leone. There would be benefits to expanding this to explore factors contributing to distress amongst young people and children, building on the work conducted by Efevbera and Betancourt in 2008 and 2010 [1].

## Conclusion

This paper examined the social determinants of psychological distress among the adult population of Sierra Leone. Three factors were identified to be the greatest contributors to psychological distress: family conflict, inability to afford basic needs, and the end of a marriage or relationship. The study also identified key differences in experiences of men and women in Sierra Leone. Factors predicting men’s psychological distress included physical health issues, financial issues, and illiteracy. For women, the predicting factors were inability to afford basic needs, poor health, the end of a marriage or relationship, and family conflict. The findings of this study indicate that interventions on enhancing income-generating and employment opportunities, promoting access to education, and family strengthening have the potential to promote mental health in Sierra Leone.

## Supplementary Information

Below is the link to the electronic supplementary material.

Supplementary file 1 (DOCX 39 KB)

## Data Availability

The datasets generated and analysed during the current study are available from the corresponding author on reasonable request.
