# Supplementary material for: Social determinants of psychological distress in Sierra Leone
Source: Soc Psychiatry Psychiatr Epidemiol. 2022 Apr 19;57(9):1781–93. doi: 10.1007/s00127-022-02278-y (PMC9375741; doi:10.1007/s00127-022-02278-y)
Supplement: Supplementary file 1 — (DOCX 39 KB) [file 127_2022_2278_MOESM1_ESM.docx]

**Social determinants of psychological distress in Sierra Leone**

Soc Psychiatry Psychiatr Epidemiol

Kanykey Jailobaeva – corresponding author

NIHR Global Health Research Unit on Health in Situations of Fragility, Institute for Global Health and Development, Queen Margaret University, Edinburgh, EH21 6UU, UK.

Email: [kjailobaeva@qmu.ac.uk](mailto:kjailobaeva@qmu.ac.uk)

[https://orcid.org/0000-0002-1316-8449](https://orcid.org/0000-0002-1316-8449?lang=en)

Other authors:

Rebecca Horn, Stella Arakelyan, Karin Diaconu, Ajaratu Kamara, Alastair Ager

**Annex 1: Outcomes of multicollinearity test**

| **Variables**  **All respondents** | **SLPD scale** | | **Subscale 1** | | **Subscale 2** | | **Subscale 3** | |
| --- | --- | --- | --- | --- | --- | --- | --- | --- |
|  | **Tolerance** | **VIF** | **Tolerance** | **VIF** | **Tolerance** | **VIF** | **Tolerance** | **VIF** |
| Someone using physical violence towards you | 0.879 | 1.138 | 0.880 | 1.137 | 0.860 | 1.163 | 0.881 | 1.135 |
| Lack of safety in the community | 0.897 | 1.114 | 0.904 | 1.107 | 0.892 | 1.121 | 0.903 | 1.107 |
| Loss of property or money | 0.897 | 1.115 | 0.900 | 1.111 | 0.886 | 1.129 | 0.899 | 1.112 |
| Severe sickness or injury | 0.670 | 1.492 | 0.672 | 1.488 | 0.663 | 1.508 | 0.672 | 1.488 |
| Loss/death of a loved one | 0.907 | 1.103 | 0.911 | 1.097 | 0.896 | 1.116 | 0.907 | 1.102 |
| Disappointment in job/college/business | 0.871 | 1.148 | 0.875 | 1.143 | 0.857 | 1.168 | 0.878 | 1.139 |
| Family conflict | 0.841 | 1.189 | 0.842 | 1.188 | 0.831 | 1.204 | 0.842 | 1.188 |
| Being unable to afford basic needs | 0.776 | 1.289 | 0.782 | 1.279 | 0.763 | 1.311 | 0.781 | 1.280 |
| End of a marriage or relationship | 0.868 | 1.152 | 0.870 | 1.149 | 0.843 | 1.186 | 0.883 | 1.132 |
| Perceiving health as poor | 0.509 | 1.964 | 0.519 | 1.927 | 0.503 | 1.990 | 0.517 | 1.935 |
| Physical health problems limiting daily work | 0.523 | 1.912 | 0.525 | 1.906 | 0.513 | 1.951 | 0.525 | 1.904 |
| Perceived difficulty to manage financially | 0.787 | 1.271 | 0.803 | 1.245 | 0.740 | 1.350 | 0.795 | 1.258 |
| Gender (ref male) | 0.689 | 1.452 | 0.709 | 1.410 | 0.635 | 1.575 | 0.703 | 1.423 |
| Employment (ref employed) | 0.855 | 1.170 | 0.871 | 1.148 | 0.831 | 1.203 | 0.868 | 1.152 |
| Marital status (ref married/ living with partner – (multiple-partner relationship)) | | | | | | | | |
| Never married | 0.508 | 1.968 | 0.555 | 1.802 | 0.374 | 2.672 | 0.549 | 1.823 |
| Separated/divorced/widowed | 0.548 | 1.826 | 0.554 | 1.805 | 0.510 | 1.961 | 0.552 | 1.813 |
| Married/ living with partner – (single-partner relationship) | 0.434 | 2.302 | 0.437 | 2.287 | 0.432 | 2.313 | 0.437 | 2.289 |
| Religious Practice (ref less frequent) | 0.937 | 1.067 | 0.939 | 1.065 | 0.909 | 1.100 | - | - |
| Lack of confidence to borrow money from a friend/family for family needs | 0.845 | 1.184 | - | - | 0.828 | 1.207 | 0.931 | 1.075 |
| Lack of confidence to talk to someone trustful about concerns | 0.845 | 1.184 | - | - | 0.862 | 1.160 | - | - |
| Cannot read (ref can read) | 0.837 | 1.194 | - | - | 0.312 | 3.200 | - | - |
| Age | - | - | - | - | 0.519 | 1.926 | - | - |
| Lack of community infrastructure | - | - | - | - | 0.844 | 1.185 | - | - |
| Education (ref above primary education) | | | | | | | | |
| No education | - | - | - | - | 0.265 | 3.769 | - | - |
| Primary Education | - | - | - | - | 0.484 | 2.065 | - | - |
| Responsibilities over dependents aged below 18 (ref above 11 children) | | | | | | | | |
| No children | - | - | - | - | 0.395 | 2.534 | - | - |
| 1 – 5 children | - | - | - | - | 0.490 | 2.041 | - | - |
| 6 – 10 children | - | - | - | - | 0.591 | 1.692 | - | - |
| **Male respondents** | **SLPD scale** | | **Subscale 1** | | **Subscale 2** | | **Subscale 3** | |
|  | **Tolerance** | **VIF** | **Tolerance** | **VIF** | **Tolerance** | **VIF** | **Tolerance** | **VIF** |
| Loss of property or money | 0.897 | 1.114 | 0.884 | 1.132 | 0.879 | 1.138 | 0.920 | 1.087 |
| End of a marriage or relationship | 0.822 | 1.216 | 0.819 | 1.221 | 0.829 | 1.206 | 0.936 | 1.069 |
| Disappointment in job/college/business | 0.830 | 1.205 | 0.830 | 1.205 | 0.815 | 1.226 | 0.846 | 1.182 |
| Family conflict | 0.907 | 1.103 | 0.901 | 1.110 | 0.902 | 1.108 | 0.903 | 1.107 |
| Severe sickness or injury | 0.662 | 1.511 | 0.661 | 1.514 | 0.637 | 1.570 | 0.660 | 1.515 |
| Being unable to afford basic needs | 0.770 | 1.298 | 0.765 | 1.308 | 0.768 | 1.302 | 0.773 | 1.294 |
| Loss/death of a loved one | 0.924 | 1.083 | 0.922 | 1.085 | 0.915 | 1.093 | 0.925 | 1.081 |
| Perceived difficulty to manage financially | 0.883 | 1.132 | 0.880 | 1.136 | 0.826 | 1.210 | 0.873 | 1.146 |
| Perceiving health as poor | 0.575 | 1.739 | 0.575 | 1.739 | 0.543 | 1.843 | 0.583 | 1.716 |
| Perceived difficulty to manage financially | 0.519 | 1.928 | 0.518 | 1.930 | 0.512 | 1.952 | 0.518 | 1.931 |
| Lack of safety in the community | 0.959 | 1.043 | 0.958 | 1.044 | - | - | - | - |
| Marital status (ref married/ living with partner – (multiple-partner relationship)) | | | | | | | | |
| Never married | 0.319 | 3.135 | 0.318 | 3.141 | 0.287 | 3.490 | - | - |
| Separated/divorced/widowed | 0.411 | 2.436 | 0.410 | 2.437 | 0.407 | 2.455 | - | - |
| Married/ living with partner – (single-partner relationship) | 0.255 | 3.914 | 0.255 | 3.916 | 0.251 | 3.990 | - | - |
| Religious Practice (ref less frequent) | 0.907 | 1.102 | 0.907 | 1.103 | - | - | - | - |
| Someone using physical violence towards you | - | - | 0.940 | 1.064 | - | - | - | - |
| Age | - | - | - | - | 0.661 | 1.512 | - | - |
| Education (ref above primary education) | - | - | - | - |  |  | - | - |
| No education | - | - | - | - | 0.271 | 3.691 | - | - |
| Primary Education | - | - | - | - | 0.480 | 2.081 | - | - |
| Cannot read (ref can read) | - | - | - | - | 0.296 | 3.377 | - | - |
| Lack of confidence to talk to someone trustful about concerns | - | - | - | - | 0.949 | 1.054 | - | - |
| Lack of community infrastructure | - | - | - | - | - | - | 0.902 | 1.109 |
| **Female respondents** | **SLPD scale** | | **Subscale 1** | | **Subscale 2** | | **Subscale 3** | |
|  | **Tolerance** | **VIF** | **Tolerance** | **VIF** | **Tolerance** | **VIF** | **Tolerance** | **VIF** |
| Someone using physical violence towards you | 0.855 | 1.169 | 0.862 | 1.160 | 0.838 | 1.194 | 0.856 | 1.168 |
| Loss of property or money | 0.907 | 1.103 | 0.914 | 1.094 | 0.905 | 1.105 | 0.915 | 1.093 |
| Severe sickness or injury | 0.645 | 1.551 | 0.639 | 1.564 | 0.636 | 1.572 | 0.648 | 1.543 |
| Loss/death of a loved one | 0.878 | 1.139 | 0.882 | 1.134 | 0.875 | 1.143 | 0.894 | 1.119 |
| End of a marriage or relationship | 0.860 | 1.163 | 0.885 | 1.130 | 0.855 | 1.170 | 0.881 | 1.135 |
| Being unable to afford basic needs | 0.758 | 1.319 | 0.765 | 1.308 | 0.747 | 1.338 | 0.786 | 1.272 |
| Family conflict | 0.806 | 1.240 | 0.823 | 1.214 | 0.797 | 1.255 | 0.814 | 1.228 |
| Perceived difficulty to manage financially | 0.503 | 1.989 | 0.504 | 1.984 | 0.500 | 2.000 | 0.533 | 1.876 |
| Perceiving health as poor | 0.468 | 2.137 | 0.466 | 2.147 | 0.457 | 2.186 | 0.471 | 2.121 |
| Perceived difficulty to manage financially | 0.795 | 1.259 | 0.809 | 1.236 | 0.758 | 1.320 | 0.778 | 1.286 |
| Lack of safety in the community | 0.893 | 1.119 | 0.918 | 1.089 | 0.879 | 1.137 | - | - |
| Disappointment in job/college/business | 0.923 | 1.083 | 0.928 | 1.078 | 0.905 | 1.105 | - | - |
| Lack of confidence to borrow money from a friend/family for family needs | 0.930 | 1.075 | - | - | 0.876 | 1.141 | 0.952 | 1.050 |
| Employment (ref employed) | 0.822 | 1.217 | 0.866 | 1.155 | 0.799 | 1.252 |  |  |
| Marital status (ref married/ living with partner – (multiple-partner relationship)) | | | | | | | | |
| Never married | 0.698 | 1.432 | - | - | 0.561 | 1.782 | 0.654 | 1.530 |
| Separated/divorced/widowed | 0.637 | 1.570 | - | - | 0.630 | 1.588 | 0.649 | 1.541 |
| Married/ living with partner – (single-partner relationship) | 0.623 | 1.605 | - | - | 0.620 | 1.613 | 0.620 | 1.612 |
| Lack of confidence to talk to someone trustful about concerns | - | - | - | - | 0.892 | 1.121 | - | - |
| Lack of community infrastructure | - | - | - | - | 0.873 | 1.145 | - | - |
| Cannot read (ref can read) | - | - | - | - | 0.845 | 1.184 | - | - |
| Responsibilities over dependents aged below 18 (ref above 11 children) | | | | | | | | |
| No children | - | - | - | - | 0.608 | 1.645 | - | - |
| 1 – 5 children | - | - | - | - | 0.566 | 1.768 | - | - |
| 6 – 10 children | - | - | - | - | 0.614 | 1.628 | - | - |
